# Persistent Retinal Microvascular Impairment in COVID-19 Bilateral Pneumonia at 6-Months Follow-Up Assessed by Optical Coherence Tomography Angiography

**DOI:** 10.3390/biomedicines9050502

**Published:** 2021-05-02

**Authors:** Valentina Bilbao-Malavé, Jorge González-Zamora, Manuel Saenz de Viteri, Miriam de la Puente, Elsa Gándara, Anna Casablanca-Piñera, Claudia Boquera-Ventosa, Javier Zarranz-Ventura, Manuel F. Landecho, Alfredo García-Layana

**Affiliations:** 1 Department of Opthalmology, Clínica Universidad de Navarra, 31008 Pamplona, Spain; vbilbao@unav.es (V.B.-M.); jgzamora@unav.es (J.G.-Z.); mdelapuentec@unav.es (M.d.l.P.); egandararod@unav.es (E.G.); aglayana@unav.es (A.G.-L.); 2 Institut Clínic de Oftalmología (ICOF), Hospital Clínic de Barcelona, 08028 Barcelona, Spain; casablancanna@gmail.com (A.C.-P.); boquera.claudia@gmail.com (C.B.-V.); zarranz@clinic.cat (J.Z.-V.); 3 Institut de Investigacions Biomediques August Pi i Sunyer (IDIBAPS), 08036 Barcelona, Spain; 4 COVID-19 Unit, Clínica Universidad de Navarra, 31008 Pamplona, Spain; mflandecho@unav.es; 5 Department of Internal Medicine, Clínica Universidad de Navarra, 31008 Pamplona, Spain

**Keywords:** SARS-COV-2, COVID-19, coronavirus, retina, microvascular, cotton wool spots, optical coherence tomography, OCT, optical coherence tomography angiography, OCTA

## Abstract

The purpose of this study was to evaluate the long-term evolution of retinal changes in COVID-19 patients with bilateral pneumonia. A total of 17 COVID-19 patients underwent retinal imaging 6 months after hospital discharge with structural optical coherence tomography (OCT) and optical coherence tomography angiography (OCTA). The parafoveal retinal nerve fiber layer (RNFL) and ganglion cell layer (GCL) were significantly thinner in COVID-19 patients at 6 months compared to 0 months (*p* = <0.001 in both cases). In the optic nerve analysis, a significantly thinner RNFL was observed (*p* = 0.006) but persisted significantly thickened, compared to controls (*p* = 0.02). The vascular density (VD) at 6 months persisted significantly decreased when compared to the control group, and no significant differences were found with the 0 months evaluation; in addition, when analyzed separately, women showed a worsening in the VD. Moreover, a significantly greater foveal area zone (FAZ) (*p* = 0.003) was observed in COVID-19 patients at 6 months, compared to 0 months. The cotton wool spots (CWSs) observed at baseline were no longer present at 6 months, except for one patient that developed new ones. This study demonstrates that some of the previously known microvascular alterations resulting from COVID-19, persist over time and are still evident 6 months after hospital discharge in patients who have suffered from bilateral pneumonia.

## 1. Introduction

First reported in Wuhan, China, in December 2019, the current coronavirus outbreak has rapidly spread worldwide causing a high number of deaths. Coronavirus 19 disease (COVID-19) is caused by severe acute respiratory syndrome coronavirus 2 (SARS-COV-2), a novel betacoronavirus that causes a life-threatening infection with an acute respiratory distress syndrome, associated with a prothrombotic state and a multiorgan failure [1,2]. From the ocular perspective, different signs and symptoms have been described in patients with COVID 19, more commonly in those suffering from severe pneumonia [3]. Retinal involvement has been reported, including retinal hemorrhages, cotton wool spots (CWSs), dilated veins, and increased vascular tortuosity [4,5,6,7,8,9,10,11,12].

In patients with severe COVID-19 multiple organ failure, microvascular endothelial injury, combined with cytokine overproduction leading to ischemia with consequent inflammation, tissue edema, and prothrombotic state, has been hypothesized as the key factor [2,13,14]. In vivo noninvasive assessment of microvascular function can be analyzed in the retina by optical coherence tomography angiography (OCTA), a novel technology capable of quantifying blood flow in the different capillary plexuses of the retina: the superficial capillary plexus (SCP), the deep capillary plexus (DCP), and the choriocapillaris (CC) [13]. In a previous study, we used OCTA to assess the retinal and choroidal vasculature in patients with bilateral pneumonia due to SARS-COV-2 that required hospital admission. We found a decreased vascular density (VD) and enlarged foveal avascular zone (FAZ) in the foveal and parafoveal retinal vascular network of COVID-19 patients, compared to a cohort of age and sex-matched healthy controls [9]. Other authors have also found reduced retinal VD in patients with recent COVID 19 and have suggested a correlation with the severity of the disease [10,12] or the levels of D-dimer [14]. Moreover, histopathological analyses of eyes from COVID 19 positive patients have confirmed the presence of vascular abnormalities consistent with ocular vein occlusions and reduced vascular densities [15].

In other organs, the epidemiological and clinical characteristics, pathogenesis, and complications of patients with COVID-19 at the acute phase have also been described [16,17]; however, many patients remain symptomatic later on. Some persisting symptoms such as fatigue and dyspnea [16,17], impaired pulmonary function [16,17], and chest image abnormalities [18] were reported in patients following hospital discharge, with a higher proportion of women affected in comparison with men [19,20,21,22]. However, the long-term consequences of the disease remain unclear and only a few studies have been published with the longest follow-up duration of 6 months after hospital discharge [18,19,20,23,24,25]. Furthermore, only a few studies have reported the extrapulmonary organ manifestations that could persist after damage in the acute stage or are developed after discharge [26,27,28], but no long-term microvascular abnormalities have been reported yet. For this reason, we decided to perform a new analysis in our cohort of COVID-19 patients 6 months after the initial assessment to describe the long-term microvascular retina alterations and investigate if the changes were more severe in women.

## 2. Materials and Methods

### 2.1. Study Design and Ethics Approval

We performed a cross-sectional case–control and a prospective longitudinal cohort study. As described in our previous paper [9], the cases’ cohort consisted of a series of COVID-19 bilateral pneumonia patients, with at least one SARS-COV-2 positive polymerase chain reaction (PCR) test, admitted at the Clínica Universidad de Navarra (Pamplona, Spain) during March and April 2020. All patients were invited to participate in the study 14 days after hospital discharge and 6 months later to repeat the ocular examination. At the moment of ocular examination, all patients were asymptomatic and had negative results from oral and nasopharyngeal swabs and positive antibodies. For the 6 months case–control study, we compared the data obtained 6 months later with the same age- and sex-matched controls randomly selected (1:1) in our previous study [9] and for the 0–6 months prospective cohort study, we compared the data from the first examination with the data obtained 6 months later.

This project was approved by the Institutional Review Board of Clínica Universidad de Navarra (study code 2020.224 approved on 9 November 2020), and age- and sex-matched controls were selected from a large OCTA database collected in a previous research project (HCB/2016/0216 approved on 16 December 2016). This study followed the tenets set forth in the Declaration of Helsinki. Written informed consent was obtained for all participants.

### 2.2. Inclusion and Exclusion Criteria

**Figure 1:**
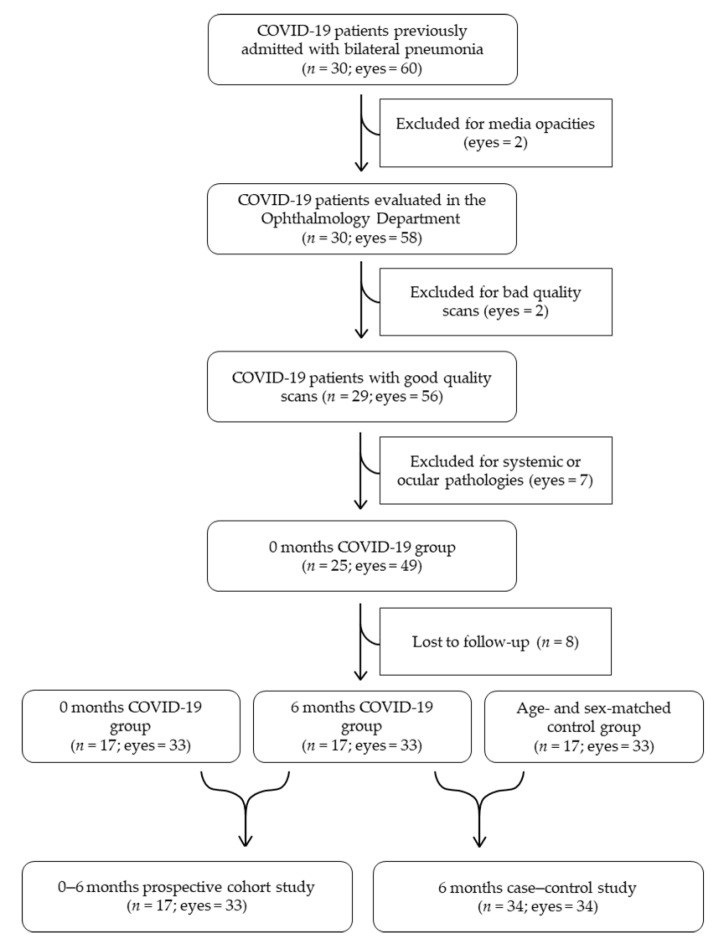
Consolidated standard for reporting trials (CONSORT)-style flow chart describing included and excluded eyes in both the case–control study and the 0–6 months prospective cohort study.

As described in our previous paper [9], all the patients presenting media opacities such as cataract and vitreous hemorrhages, poor quality scans, or concomitant systemic or ocular pathologies such as diabetes mellitus, glaucoma, high myopia, fovea plana, and age-related macular degeneration were excluded from analysis, resulting on a total of 25 patients (eyes = 49). Six months later we contacted these 25 patients and invited them to repeat the ocular examination; 17 patients (eyes = 33) agreed to return. To avoid a potential risk of bias due to the bilaterality of cases, only one eye of each of these patients was randomly selected and included in the 6-month case–control study (*n* = 34; eyes = 34). In the 0–6 months prospective cohort study both eyes from each participant were used on the analysis (*n* = 17; eyes = 33). A consolidated standard for reporting trials (CONSORT)-style flow chart describing included and excluded eyes in both studies (case–control study and 0–6 months prospective study) is presented in Figure 1.

### 2.3. Ocular Examination, OCT, and OCTA Imaging Acquisition Protocols

The same ocular examination performed at baseline was repeated in all participants 6 months later and included best-corrected visual acuity (BCVA), slit-lamp biomicroscopy, intraocular pressure measurement, retinal fundus examination, fundus retinographies, OCT, and OCTA scans (DRI OCT Triton SS-OCT Angio, Topcon Medical Systems, Inc. Oakland, NJ, USA). Images were captured using the same scanning protocols employed in the first study (3D Wide 12 × 9 mm and 3D Disc 6 × 6 mm for structural OCT images, and 4.5 × 4.5 mm and 6 × 6 mm for OCTA).

The retinal stratus and parameters analyzed were total retina, retinal nerve fiber layer (RNFL), ganglion cell layer (GCL), and choroid thicknesses as delineated by the boundaries automatically defined by the built-in segmentation software in the commercial device. Optic nerve head RNFL thickness was measured in each of the four quadrants using a radial scan centered on the optic nerve head and presented as mean RNFL.

OCTA parameters evaluated were vessel density (VD) in the superficial capillary plexus (SCP) and the deep capillary plexus (DCP). Mean VD was calculated as the average value obtained in the fovea and parafoveal area, as previously described [9]. The foveal avascular zone (FAZ) area was manually delineated on the SCP by two independent graders, encompassing the central fovea where there were no clear and demarcated vessels seen on the OCTA. Both, structural OCT and OCTA parameters were measured using the early treatment diabetic retinopathy (ETDRS) grid centered in fovea by fixation.

### 2.4. Statistical Analysis

To estimate the minimum sample size for adequate study power, we considered an alpha error of 0.05 and a power of 90%. For the anticipated incidence, we used the 33% incidence of CWSs reported by Marinho et al. [7], which was the only study that referred to COVID-19 retinal changes at the time of the study design. After this calculation, the minimum size recommended was 24 patients for each group, and we recruited 25 after the exclusion criteria.

The description of quantitative variables was performed using the mean and the standard error of the mean (SEM). The Kolmogorov–Smirnov test was used to assess the normality of distributions. The quantitative variables that followed a normal distribution were studied with the independent Student’s *t*-test in the case–control study and the paired *t*-test in the 0–6 months prospective cohort study. In the case of the non-normally distributed variables, Mann–Whitney U test was used in the case–control study, and Wilcoxon signed-rank test in the 0–6 months prospective cohort study. For the manually measured FAZ area, the intraclass correlation coefficient was used to evaluate the agreement between two independent graders (V.B.-M. and J.G.-Z.) and an excellent agreement between them was found (ICC = 0.98); hence, the mean of these two measurements was employed for further analysis. A gender subanalysis was performed, mean OCTA values at 0 and 6 months were compared for women and men separately, and then the ratio between the values at 6 and 0 months (6/0 m ratio) was calculated for women and men to compare both of them.

For all the tests, *p* values < 0.05 were considered statistically significant. The Bonferroni method was used to correct for multiple comparisons. Statistical analyses were performed using GraphPad Prism software version 8.0.1 (GraphPad Software Inc., San Diego, CA, USA) and SPSS software version 25 (SPSS Inc., Chicago, IL, USA).

## 3. Results

The cohort of COVID-19 bilateral pneumonia cases analyzed in both the 6 months case–control study and the 0–6 months prospective cohort study, included 17 eyes from 17 patients. This group was composed of 9 right eyes and 8 left eyes and included 8 women and 9 men. The mean age of the cases and controls was 58.0 ± 2.7 and 57.2 ± 2.7 years old, respectively (*p* = 0.85). The mean refraction of the cases and controls was −0.07 ± 0.56 diopters (D) and −0.35 D ± 0.52 D (*p* = 0.918). The mean axial length (AL) of the control group was 23.68 ± 0.66 mm. Due to COVID-19 restrictions at examination time, AL could not be assessed in the cases, but none of them had undergone refractive surgery and only one was pseudophakic; therefore, extreme AL was unlikely to be present. Seven out of 17 patients (two men and five women) reported at least one symptom related to COVID-19 3 months after hospital discharge, particularly dyspnea, alopecia, hyposmia, ageusia, fatigue, muscle pain, and emotional lability.

### 3.1. Case–Control Study (6 Months)

In the structural OCT analysis, a significantly thicker mean optic nerve RNFL was observed in COVID-19 cases at 6 months evaluation, compared to controls (111.5 ± 3.79 vs. 102.23 ± 4.50, *p* = 0.02) but this significance was lost after the Bonferroni adjustment (*p* = 0.16). In the SCP, a significantly lower VD was observed in COVID-19 patients at 6 months evaluation, compared to controls in the fovea (14.40 ± 1.14 vs. 29.48 ± 2.62, *p* < 0.001). In this plexus, a greater FAZ area was observed in COVID-19 patients at 6 months, compared to controls (315.198 ± 32.94 vs. 191.44 ± 33.65, *p* = 0.004). In the DCP, a significantly lower VD was observed in the foveal area of COVID-19 patients at 6 months evaluation, compared to controls (13.13 ± 1.18 vs. 18.83 ± 1.59, *p* < 0.02), although this was lost after the Bonferroni correction (*p* = 0.16). No significant differences were found in the SCP and DCP VD of the parafoveal area (Table 1).

### 3.2. Prospective Cohort Study (0–6 Months)

In the structural OCT analysis, a significantly thicker central retina was observed in COVID-19 patients at 6 months, compared to baseline evaluation (246.7 vs. 244.2, difference of 2.54 ± 0.43, *p* = <0.001). The parafoveal RNFL was significantly thinner in COVID-19 patients at 6 months, compared to 0 months (246.7 vs. 244.2, difference of −0.88 ± 0.04, *p* = <0.001). The GCL analysis showed that the parafoveal GCL was significantly thinner in COVID-19 patients at 6 months, compared to 0 months (88.05 vs. 88.77, difference of −0.72 ± 0.13, *p* = <0.001). Central choroid thickness was significantly thicker in COVID-19 patients at 6 months, compared to 0 months (249.8 vs. 243.1, difference of 6.76 ± 2.68, *p* = 0.017), but this significance was lost after the Bonferroni adjustment (*p* = 0.17). In the optic nerve analysis, a significantly thinner mean RNFL was observed in COVID-19 cases at 6 months evaluation compared to 0 months (111.03 vs. 113.75, difference of −1.63 ± 0.61, *p* = 0.006), although this significance was lost after Bonferroni adjustment (*p* = 0.06). (Table 2).

In the OCTA analysis, no significant differences in VD were observed in the foveal and parafoveal SCP analysis in COVID-19 patients at 6 months evaluation, compared to 0 months (14.26 vs. 14.66, *p* = 0.179 and 40.26 vs. 40.74, *p* = 0.135, respectively). In this plexus, the FAZ area was significantly greater in COVID-19 patients at 6 months than at 0 months evaluation (321.4 vs. 308.5, difference of 12.8 ± 3.9, *p* = 0.003). In the DCP, no significant differences were found in VD in the foveal area of COVID-19 patients at 6 months, compared to 0 months, whereas a significantly lower VD was observed in the parafoveal area (41.52 vs. 42.17, difference of -0.60 ± 0.22, *p* = 0.01) that was lost after Bonferroni correction (*p* = 0.1) (Table 2).

### 3.3. Gender Analysis at 0 and 6 Months

**Figure 2:**
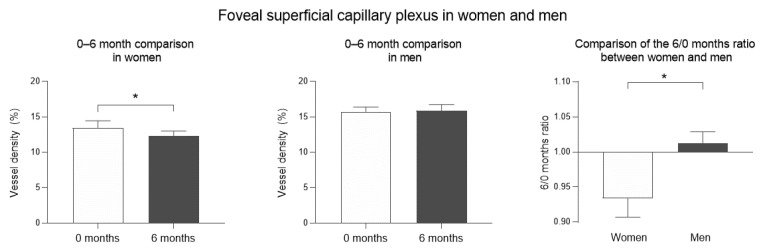
Optical coherence tomography angiography (OCTA) analysis in men and women. Superficial capillary plexus (SCP) analysis. Mean vessel density (%) of the foveal and parafoveal regions. Error bars correspond to the SEM. * *p* < 0.05.

In COVID-19 patients, a comparison analysis between women and men OCTA values at 0 and 6 months was performed (Table 3). In the OCTA analysis, women showed a significantly decreased VD in the foveal SCP at 6 months, compared to 0 months evaluation (12.33 vs. 13.47, difference of −1.13 ± 0.81, *p* = 0.013) that was lost after Bonferroni correction (*p* = 0.065), while men did not show statistically significant differences but seemed to exhibit an opposite tendency. In fact, a significant difference was demonstrated when women and men were compared (*p* = 0.013), although it was lost after Bonferroni adjustment (*p* = 0.065) (Figure 2). The FAZ area was significantly increased in women (383.4 vs. 364.9, difference of 18.63 ± 6.35, *p* = 0.004) and men (269.8 vs. 261.7, difference of 8.18 ± 4.86, *p* = 0.039) at 6 months, compared to 0 months, although in men, this difference was lost after Bonferroni correction (*p* = 0.195), and no statistically significant differences were found when comparing women and men. The analysis of parafoveal SCP and foveal and parafoveal DCP vascular density showed no statistically significant differences in any of the performed comparisons.

### 3.4. Cotton Wool Spots Analysis

**Figure 3:**
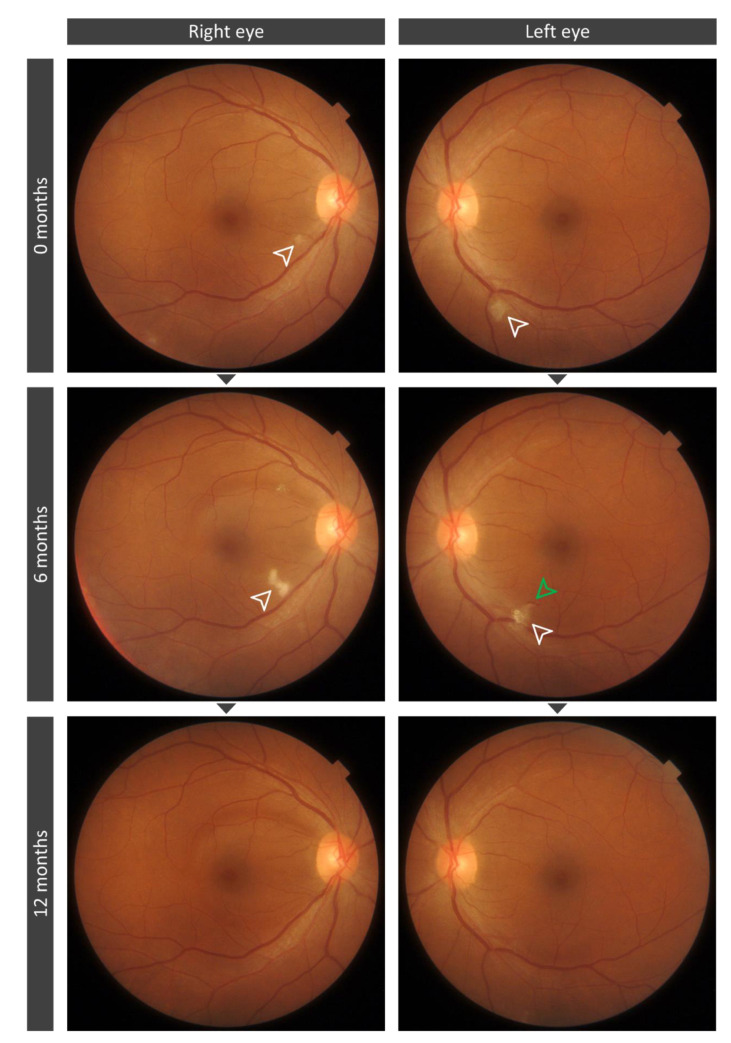
Composite fundus retinographies from both eyes of a 45-year-old woman with COVID-19 at 0 months after COVID-19 hospital discharge, 6 months later, and 12 months later. In this case, the patient presented cotton wool spots (CWSs) in both eyes in the first evaluation that were not present 6 months later, but new CWSs and a microhemorrhage were visible. Top row: Fundus retinography of the right and left eye at first evaluation. Middle row: Fundus retinography of the right and left eye at 6 months evaluation. Bottom row: Fundus retinography of the right and left eye at 9 months evaluation. White arrowheads indicate the CWSs. Green arrowhead indicates the microhemorrhage.

**Figure 4:**
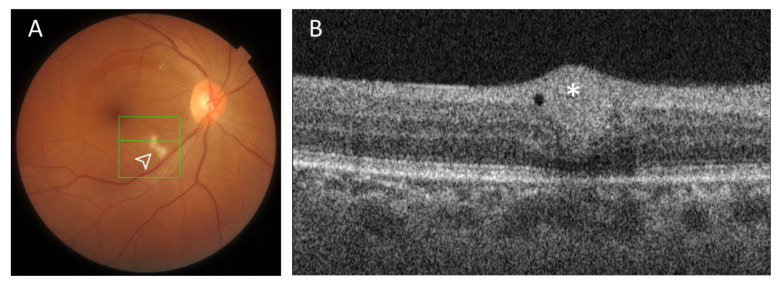
Right eye of a 45-year-old woman showing cotton wool spots (white arrowhead) 6 months after COVID-19 hospital discharge. (**A**). Right retinal color fundus photography of an isolated cotton wool spots (CWSs). (**B**). B-scan optical coherence tomography reveals localized swelling of the nerve fiber layer at the location of the CWSs (white asterisk).

As in our previous paper, when present, CWSs were located outside the analyzed areas, and therefore, the possibility of projection artifacts in the OCT was discarded. As mentioned in our previous paper [9], at 0 months, five patients (eyes = 6) presented CWSs on the posterior pole (20%), including two women and three men with a mean age of 53.83 ± 1.60 years old. Two patients had obesity and hypertension, three had hypercholesterolemia, and one had diabetes mellitus, but none of them had background features of any retinopathy (diabetic, hypertensive, or anemic retinopathy). Interestingly, no CWSs were observed at 6 months evaluation in four out of these five patients (eyes = 4). However, in the remaining patient (eyes = 2), a 45-year-old woman of Latin American origin with obesity and hypertension, the CWSs found in the first evaluation were no longer present, but new CWSs developed bilaterally in different locations of the posterior pole and also an additional microhemorrhage was present in one of the study eyes. At 12 months of evaluation, these CWSs disappeared (Figure 3 and Figure 4). This patient tested negative for HIV.

In the structural OCT and OCTA analysis, when comparing COVID-19 patients with CWSs at 6 months with controls and at 6 months with 0 months, no statistically significant differences were found.

## 4. Discussion

This study demonstrates that some of the previously known microvascular alterations resulting from COVID-19 persist over time and are still evident 6 months after hospital discharge in patients who have suffered from bilateral pneumonia, showing a persistently decreased VD and even a progressive decreased VD when women were assessed separately.

Most COVID-19 survivors had good physical recovery from their illness; however, several articles reported that a large number of patients had continued symptomatology months after their hospital discharge, with up to 33% and 40% suffering decreased mental health [29] and chronic fatigue [30]_,_ respectively, and over 50% presenting memory and smell loss 3 months later [27]. In fact, 41% of our patients presented symptoms related to COVID-19 3 months later.

During and shortly after the acute phase of the infection most of the organ dysfunctions have been attributed to microvascular angiopathy [31]. Therefore, following the microvascular changes over time seems relevant during the recovery phase of the disease. In this study, when we analyzed the VD and FAZ area in the first evaluation at 0 months, we found a decreased VD and enlarged FAZ area in the foveal and parafoveal vascular network of COVID-19 patients, compared to a cohort of age and sex-matched healthy controls [9]. Six months later, when comparing the OCTA analysis, no statistically significant differences were found; therefore, it appears that the changes observed have not been reversed; however, a trend toward a further decreased VD, without statistical significance, was observed. In addition, when comparing the 6 months OCTA values with age and sex-matched controls, these values continued to be significantly decreased. However, a comparison with the control group at 6 months would have been helpful to mitigate the OCTA changes that occur with normal aging.

Therefore, it seems that the retinal microvascular changes observed in the acute phase of the disease persist even 6 months after the infection, a phenomenon that could occur in other vascular plexuses in the body, especially in those closely related, such as the brain circulation, that share a common embryological origin and function. In this sense, one study reported that patients who recovered from pneumonia still exhibited brain microstructure and cerebral blood flow changes 3 months later [28], while another revealed volumetric and microstructural abnormalities in the central olfactory cortices and white matter in the recovery stages of COVID-19 in comparison with controls, suggesting the long-term consequences of neurological damage related to COVID-19 [27]. Persistent changes in other organs have also been reported in COVID-19 patients, months after discharge. Results of lung function analysis showed that a considerable proportion of patients had a pulmonary diffusion abnormality 6 months after symptom onset [30], and persistent renal dysfunctions, venous, and thromboembolic diseases were observed months after the infection [32]. However, to our knowledge, this is the first time that changes in the microvasculature have been quantified months after infection.

When comparing women and men in the OCTA, women showed a significantly decreased VD in the foveal SCP, suggesting two possible options: either a recovery process could be taking place more slowly in women or they may be getting progressively worse. However, interestingly, when comparing VD values between 0 and 6 months, women showed a significant decrease, while men showed a trend toward improvement. According to this, it appears that women could have an increased risk of worsening VD loss, in comparison with men, 6 months after COVID-19 infection. In this sense, other authors have found that women are also more likely to have persistent neurological and psychological symptoms [20,21], fatigue, post-activity polypnea, and alopecia [19].

In contrast, after 6 months of follow-up, the original significant thickening of the RNFL optic nerve head observed at 0 months had decreased; however, the greater thickness remained significant, when compared with the control group. This could mean that a process of recovery could be taking place. In this sense, in four out of five patients the CWSs observed at 0 months were no longer present 6 months later. Interestingly, in a 45-year-old woman, the CWSs that were presented in both eyes in the first evaluation were not observed 6 months later, but new CWSs and a microhemorrhage were visible. Moreover, this woman showed the greatest decrease in VD of all patients. No other possible explanation for these CWSs, other than that persistent changes related to COVID-19 infection were found in this patient, and at 12 months evaluation, they had disappeared. In a recent 3 months follow-up study [11], from five patients that presented CWSs in the convalescent phase of the disease, occasionally accompanied by an intraretinal hemorrhage, one patient developed recurring CWSs, as in our case. The mechanism by which CWSs could develop during COVID-19 recovery remains unclear; however, several hypotheses have been postulated to explain their development during the acute infection, including occlusive vasculopathy, angiotensin-converting enzyme 2 (ACE2) downregulation by SARS-COV-2, hypercoagulopathy, or an immune complex deposition in the vessel walls [11].

When comparing the structural OCT between 0- and 6-months evaluation, the GCL thickness was significantly decreased. In the first evaluation, this retinal layer was thinner in COVID-19 patients, in comparison with the control group, and therefore, it seems it is becoming thinner over time. In fact, this decrease in GCL thickness may be contributing to the RNFL thinning mentioned above since the RNFL consists of the axons of the GCL. Despite the fact that the magnitude of this change could seem small, it represents a decrease of 1% in GCL thickness over a short period of time, which cannot be explained by the normal age-related loss [33]. If this GCL thinning is confirmed, it would mean that a loss of neural tissue is happening in the eye long after the infection; therefore, a central cerebral system neuronal loss should be discarded.

We acknowledge a series of limitations in our study. First, the small sample size of COVID-19 bilateral pneumonia cases cohort as a consequence of loss to follow-up. Second, our control group included healthy volunteers, but it would have been interesting to compare our findings with post-recovery pneumonia patients. Third, baseline data of the pre-infection status of the study eyes was not available; hence, no comparison with the current status of the lesions was possible. In addition, since some of the microvascular retinal changes have not reverted completely at 6 months, we believed that a longer follow-up should be conducted in order to monitor the evolution. Another interesting study that could be carried out in the future will be to correlate the microvascular retinal changes with the severity of the disease and the histopathologic analysis of the retina in patients with COVID-19.

## 5. Conclusions

To our knowledge, this is the first study to follow the microvascular retinal changes 6 months after hospital discharge in patients with bilateral pneumonia due to COVID-19. We found a persistently decreased SCP and DCP VD and enlarged FAZ; in addition, women showed a significant worsening in the SCP VD 6 months later. The CWSs described in some patients in the first evaluation were absent after 6 months, except for a patient who showed new CWSs. Taking into account that many patients present with late symptomatology with our findings, we consider that is important to perform close surveillance of COVID-19 survivors for long-term disease sequelae, especially in patients with severe disease.

## Figures and Tables

**Table 1:** Results from the case–control study.

Mean OCT and OCTA Values	Controls	6 Months	*p* Value	Adjusted *p* Value
Foveal thickness (µm)	239.71 ± 10.44	245.58 ± 4.99	0.75	1
Central Choroid thickness (µm)	267.71 ± 19.91	246.88 ± 22.51	0.47	1
Optic Nerve RNFL thickness (µm)	102.23 ± 4.50	111.5 ± 3.79	**0.02**	0.16
Foveal SCP vascular density (%)	29.48 ± 2.62	14.40 ± 1.14	**<0.001**	**0.008**
Parafoveal SCP vascular density (%)	42.09 ± 0.59	40.51 ± 0.47	**0.07**	0.56
Superficial FAZ area (µm^2^)	191.44 ± 33.65	315.198 ± 32.94	**0.004**	**0.032**
Foveal DCP vascular density (%)	18.83 ± 1.59	13.13 ± 1.18	**0.02**	0.16
Parafoveal DCP vascular density (%)	42.61 ± 0.63	41.76 ± 0.48	0.47	1

Comparison of structural optical coherence tomography (OCT) and OCT angiography (OCTA) parameters in COVID-19 patients at 6 months and age-, sex-, and laterality-matched controls. Mean ± SEM (standard error of the mean) structural OCT and OCTA values. *p* values correspond to comparisons between subgroups. Adjusted *p* value corresponds to *p* value after Bonferroni correction. Nerve fiber layer (RNFL); superficial capillary plexus (SCP); foveal avascular zone (FAZ); deep capillary plexus (DCP). Bold values denote statistical significance at the *p* < 0.05 level.

**Table 2:** Results from the 0–6 months prospective cohort study. Comparison of structural optical coherence tomography (OCT) and OCT angiography (OCTA) parameters in COVID-19 patients at 0 and 6 months.

Mean OCT and OCTA Values	0 Months	6 Months	Difference	*p* Value	Adjusted *p* Value
Foveal thickness (µm)	244.2	246.7	2.54 ± 0.43	**<0.001**	**0.01**
Parafoveal RNFL thickness (µm)	27.66	26.87	−0.88 ± 0.04	**<0.001**	**0.01**
Parafoveal GCL thickness (µm)	88.77	88.05	−0.72 ± 0.13	**<0.001**	**0.01**
Central Choroid thickness (µm)	243.1	249.8	6.76 ± 2.68	**0.017**	0.17
Optic Nerve RNFL thickness (µm)	113.75	111.03	−1.63 ± 0.61	**0.006**	0.06
Foveal SCP vascular density (%)	14.66	14.26	−0.37 ± 0.28	0.179	1
Parafoveal SCP vascular density (%)	40.74	40.26	−0.47 ± 0.25	0.135	1
Superficial FAZ area (µm^2^)	308.57	321.43	12.85 ± 3.97	**0.003**	**0.03**
Foveal DCP vascular density (%)	13.26	12.99	−0.28 ± 0.38	0.472	1
Parafoveal DCP vascular density (%)	42.17	41.52	−0.60 ± 0.22	**0.01**	0.1

Mean ± SEM (standard error of the mean) structural OCT and OCTA values. *p* values correspond to a comparison between subgroups. Adjusted *p* value corresponds to *p* value after Bonferroni correction. Nerve fiber layer (RNFL); ganglion cell layer (GCL); superficial capillary plexus (SCP); foveal avascular zone (FAZ); deep capillary plexus (DCP). Bold values denote statistical significance at the *p* < 0.05 level.

**Table 3:** Results from the 0–6 months prospective cohort study between women and men.

Mean OCTA Values	0–6 Month Comparison in Women (*n* = 8; Eyes = 15)				0–6 Month Comparison in Men (*n* = 9; Eyes = 18)				Comparison of the Difference between Women and Men			
	0 m	6 m	*p* Value	Adj *p* Value	0 m	6 m	*p* Value	Adj *p* Value	6/0 m Ratio in Women	6/0 m Ratio in Men	*p* Value	Adj *p* Value
Foveal SCP VD (%)	13.47	12.33	**0.013**	0.065	15.65	15.89	0.573	2.865	−0.93	1.01	**0.013**	0.065
Parafoveal SCP VD (%)	50.10	49.25	0.131	0.655	47.25	46.71	0.791	3.955	−0.98	-0.99	0.708	1
Superficial FAZ area (µm^2^)	364.9	383.4	**0.004**	**0.02**	261.7	269.8	**0.039**	0.195	1.05	1.03	0.580	1
Foveal DCP VD (%)	12.07	11.25	0.131	0.655	14.26	14.44	0.791	3.955	−0.92	1.01	0.342	1
Parafoveal DCP VD (%)	42.24	41.46	0.095	0.475	42.12	41.67	0.263	1.315	−0.98	-0.99	0.478	1

Comparison of OCT angiography (OCTA) parameters in COVID-19 patients at 0 and 6 months. Mean structural OCT and OCTA values. *p* values correspond to a comparison between subgroups. Adjusted *p* value corresponds to *p* value after Bonferroni correction. Superficial capillary plexus (SCP); foveal avascular zone (FAZ); deep capillary plexus (DCP); vascular density (VD); adjusted (Adj). Bold values denote statistical significance at the *p* < 0.05 level.

## Data Availability

All data are available within the manuscript and upon request to the corresponding author.
